# Unique pathological phalangeal fusion in the chalicothere subfamily Chalicotheriinae and the interphalangeal immobilization in chalicotheres

**DOI:** 10.1007/s00114-025-02011-0

**Published:** 2025-08-18

**Authors:** Panagiotis Kampouridis, Christina Kyriakouli, Gabriel de Souza Ferreira

**Affiliations:** 1 Department of Geosciences, Eberhard Karls University of Tübingen, Tübingen, Germany; 2 Senckenberg Centre for Human Evolution and Palaeoenvironment, Tübingen, Germany

**Keywords:** Paleopathology, Mammalia, μCT scan, Ankylosis, Cenozoic

## Abstract

Chalicotheres are bizarre extinct herbivore mammals closely related to today’s rhinoceroses, tapirs and horses. The family Chalicotheriidae includes two subfamilies, the Chalicotheriinae and the Schizotheriinae. Some members of the schizotheriines form a duplex bone by fusing the proximal and the medial phalanges of the second digit of the hand. Here, we report the only known fused set of proximal and medial phalanges in a specimen of the subfamily Chalicotheriinae from the Late Miocene of Höwenegg in Germany. This specimen has been the center of some confusion regarding its identity and the nature of the fusion. In our study, we conduct a detailed comparison to chalicotheriines and schizotheriines, identifying the specimen as a chalicotheriine and attributing its fusion to a pathology. We additionally acquired CT scans of this specimen and two schizotheriines to compare the internal structure of the fused phalanges, which revealed great differences between the fusion in schizotheriines and the pathologically fused Höwenegg specimen. Furthermore, we found that both subfamilies show a trend towards immobilizing their digits, expressed in different ways, with chalicotheriines forming a notched-joint between the phalanges in some digits that hinders their movement and schizotheriines regularly fusing the phalanges.

## Introduction

Chalicotheres are an extinct peculiar group of perissodactyl mammals, closely related to extant rhinoceroses, tapirs, and horses. They exhibit a number of unusual traits, most prominently the presence of claws, instead of hooves, on both their hands and feet (Coombs 1983). These claws are deeply cloven and have greatly confused researchers in the past, as exemplified by the fact that the first ever documented chalicothere claw was attributed to a giant pangolin (Cuvier 1823). The existence of large claws is consistent with the current knowledge about their diet, which includes browsing on leaves, bark, and twigs (Schulz et al. 2007; Semprebon et al. 2011).

The Chalicotheriidae include two subfamilies, the Chalicotheriinae and the Schizotheriinae, which differ in certain features of the skull, teeth, and appendicular skeleton (Coombs 1983). In derived members of the schizotheriines the proximal and medial phalanges fuse, forming the so-called duplex bone (Coombs and Rothschild 1999). Such phalangeal fusion is very rare in the fossil record (e.g., Luo et al. 2015; Averianov 2025) and is not seen in any other member of the Chalicotheriidae. However, Zapfe (1989) described a set of fused phalanges of *Chalicotherium goldfussi* (Hö 142) from the Late Miocene of Höwenegg (Germany). Later, Fahlke and Coombs (2009) suggested that this specimen actually belongs to a schizotheriine, possibly *Metaschizotherium* or *Ancylotherium*. We found that the Höwenegg specimen (Hö 142), however, is certainly a chalicotheriine, because its morphology deviates significantly from known schizotheriine duplexes both externally and internally. The aim of this study is to redescribe specimen Hö 142, the only known case of fused proximal and medial phalanges in the chalicothere subfamily Chalicotheriinae. The specimen was μCT scanned to compare it to physiologically fused duplex bones of schizotheriines, in order to identify it as a pathological fusion. We will also show that in both subfamilies the movement of the phalanges was very restricted, while this restriction was achieved through different morphologies.

## Material and methods

Micro-computed tomography (µCT) scans were acquired for Hö 142 and two further chalicothere specimens. Specimen Hö 142 (housed at the Fürstlich Fürstenbergische Sammlungen in Donauschingen, Germany) and a pedal duplex bone of *Metaschizotherium fraasi* from the Middle Miocene of Steinheim in Germany (SMNS-P-5815) were scanned with a Nikon XT H 320 μCT scanner operated by the 3D Imaging Lab of the Eberhard Karls University of Tübingen and Senckenberg Centre for Human Evolution and Palaeoenvironment Germany (SHEP). Specimens Hö 142 and SMNS-P-5815 were scanned at 220 kV and 100 μA with a voxel size of 0.03026702 mm and 0.02873177 mm, respectively, using a copper filter of 0.25 mm thickness. A manual duplex bone of *Ancylotherium pentelicum* from the Late Miocene of Pikermi in Greece (NHMW-GEO-2019/0098/0007) was scanned with a YXLON FF35 CT scanner at the Natural History Museum in Vienna (NHMW) at 190 kV and 350 μA with a voxel size of 0.0300 mm, using a copper filter of 3 mm thickness.

The acquired CT scans of Hö 142 and SMNS-P-5815 are available on Morphosource under the Project ID 000769060. The CT scan of NHMW-GEO-2019/0098/0007 is available on the NHMW Data Repository: 10.57756/b1su1x.

## Description

**Fig. 1:**
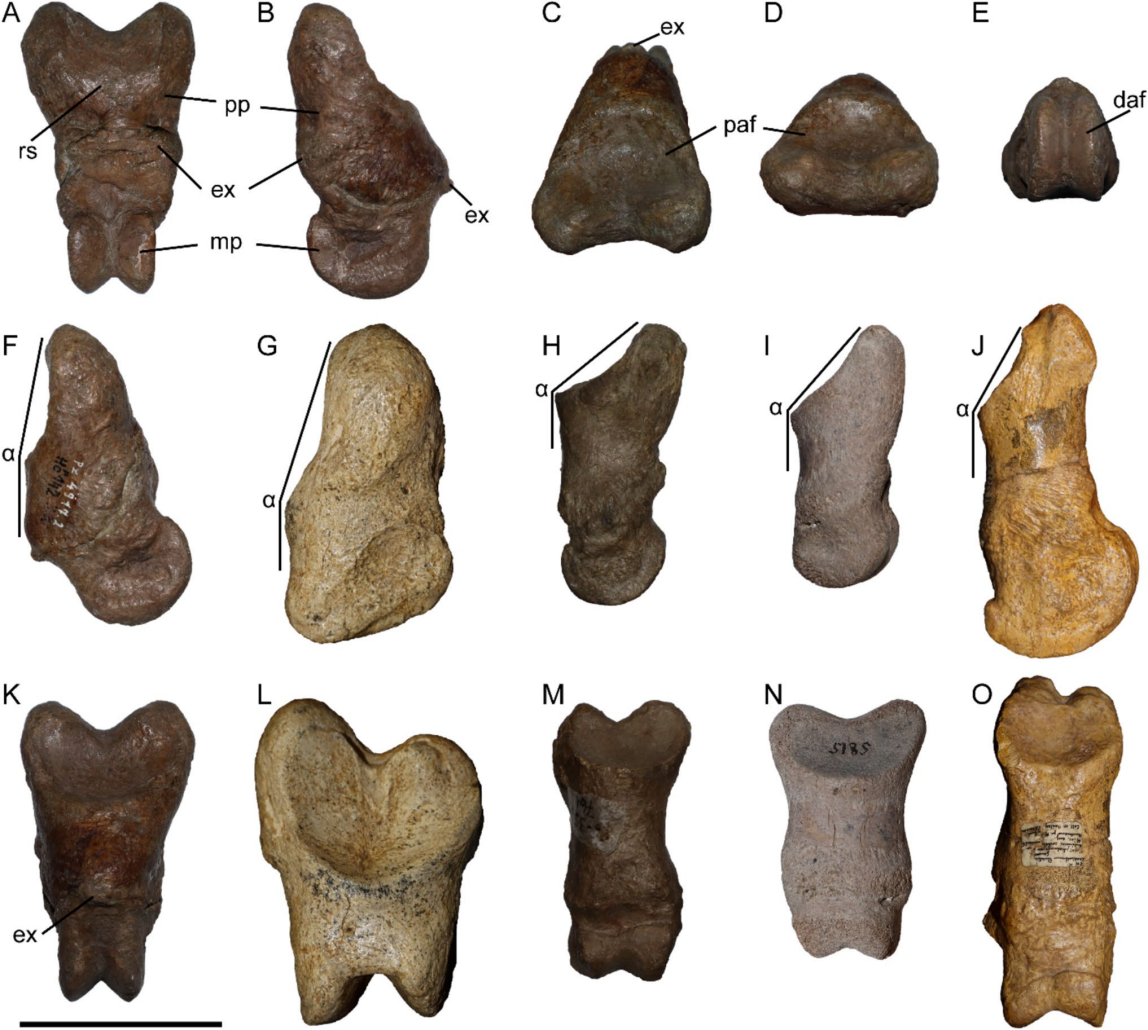
Pathologically fused proximal and medial phalanges (Hö 142) of a chalicotheriine from the Late Miocene locality of Höwenegg (Germany) in ventral (**A**), right side (**B**), proximal-dorsal (**C**), proximal (**D**), distal (**E**), left side (**F**), and dorsal (**K**) views. Comparative chalicothere phalanges in left side (**F, G, H, I, and J**) and dorsal (**K, L, M, N, and O**) views: proximal phalanx of the manus of *Chalicotherium goldfussi* (HLMD Din 3170) from the Late Miocene of Eppelsheim, Germany (**G, L**); duplex bone of the pes of *Metaschizotherium bavaricum* (NMA 86–356/761) from the Middle Miocene of Häder, Germany (**H, M**); and duplex bone of the pes of *Metaschizotherium fraasi* (SMNS-P-5815) from the Middle Miocene of Steinheim, Germany (**I, N**); duplex bone of the manus digit II of *Ancylotherium pentelicum* (MGL 106762) from the Late Miocene of Samos, Greece (**J, O**). Abbreviations: α, angle formed by the proximal articular facet and the long axis of the shaft; ex, exostosis; daf, distal articular facet of the medial phalanx; mp, medial phalanx; paf, proximal articular facet of the proximal phalanx; pp, proximal phalanx; and rs, rugose surface. Scale bar equals 5 cm for A–I, K–N, and 10 cm for J and O

Specimen Hö 142 represents a set of fused proximal and medial phalanges of the third digit (Fig. 1). The proximal articular facet for the metapodial is heart-shaped and rather shallow. The proximo-ventral border of the facet bears a prominent incision in the middle, and the distal end is rounded and blunt. The proximal facet is quite large and placed obliquely, on the dorsal side of the bone, being placed almost parallel to the proximo-distal axis of the phalanx (in Fig. 1F). On the ventral side of the bone a rugose half-moon-shaped surface can be observed proximally. Distally, the phalanges narrow down, forming an almost triangular shape. The bilateral keels of the distal articulation of the medial phalanx are symmetrical and very sharp, forming a deep valley between them.

## Comparison

Chalicotheriidae are divided into the two subfamilies Chalicotheriinae and Schizotheriinae. Specimen Hö 142 was originally assigned to the chalicotheriine *Chalicotherium goldfussi* (Zapfe 1989). Later on, Fahlke and Coombs (2009) attributed the specimen to a schizotheriine, possibly *Metaschizotherium* or *Ancylotherium*, due to the fusion, which mimics the schizotheriine duplex.

**Fig. 2:**
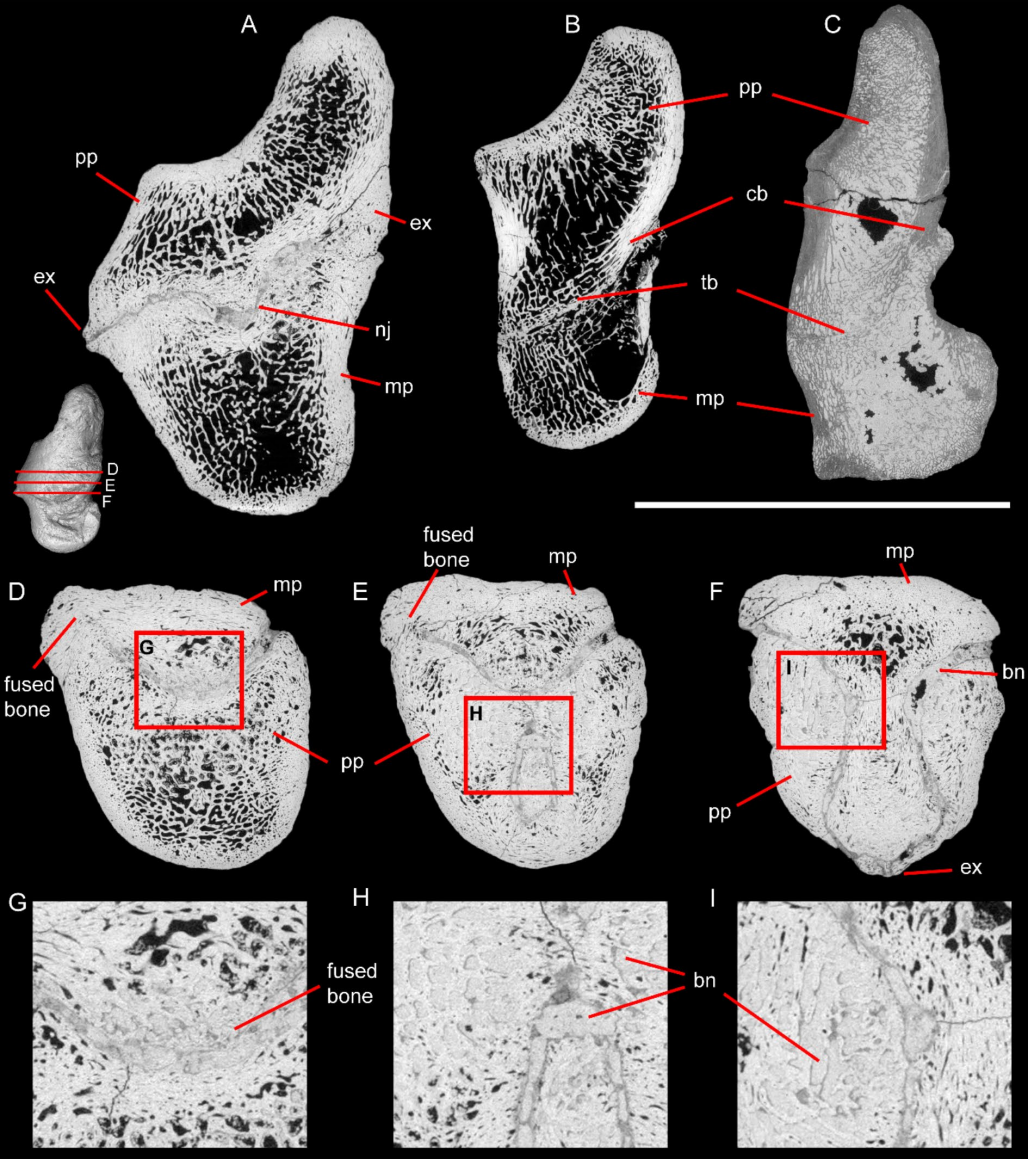
Virtual cross-sections of the pathologically fused proximal and medial phalanges (Hö 142) of a chalicotheriine from the Late Miocene of Höwenegg, Germany **(A, D–I)** and duplex bones of schizotheriines **(B, C)**. Sagittal cross-sections in the middle of Hö 142 **(A)**, the duplex bone of the pes of *Metaschizotherium fraasi* (SMNS-P-5815) from the Middle Miocene of Steinheim, Germany **(B)** and the duplex bone of the manus digit II of *Ancylotherium pentelicum* (NHMW-GEO-2019/0098/0007) from the Late Miocene of Pikermi, Greece **(C)**. Horizontal cross-sections at different positions of Hö 142 with ventral side up **(D–F)**. Magnified areas of the horizontal cross-sections of Hö 142 **(G–I)**. Abbreviations: cb, remaining cortical bone at the fusion zone of the phalanges; ex, exostosis; mp, medial phalanx; nj, notched joint; pp, proximal phalanx; bn, bone neoformation; and tb, intertwined trabecular bone at the fusion zone of the phalanges. Scale bar equals 5 cm for **A-B, D–F**, 10 cm for **C**, and 2 cm for **G–I**

The schizotheriines during the Middle to Late Miocene are represented in central Europe by the genera *Metaschizotherium* and *Ancylotherium*, while the chalicotheriines are represented by *Anisodon* and *Chalicotherium* (e.g., Zapfe 1967, 1979; Coombs 2009; Fahlke and Coombs 2009; Guérin 2012; Fahlke et al. 2013; Kampouridis et al. 2022, 2024a, b). The Höwenegg specimen differs from schizotheriines in having a proximal articular facet that is oriented almost parallel to the long axis of the shaft, whereas in schizotheriines it usually forms an acute angle (α in *Metaschizotherium* and *Ancylotherium* in Fig. 1H-J). In *Anisodon grande* and *Chalicotherium goldfussi*, the proximal facet is also placed subparallel to the long axis of the phalanx (Fig. 1G; Zapfe 1979). The proximal phalanx of Hö 142 also differs from schizotheriines in having a single rugose surface proximally on the ventral side for the attachment of muscles (Fig. 1A). In schizotheriines, there are usually two well-separated tuberosities in this area (Coombs 1978; Kampouridis et al. 2023), whereas in the chalicotheriines *Anisodon* and *Chalicotherium*, there is a continuous rugose area (Zapfe 1979). Additionally, in schizotheriine duplexes (Fig. 1H–J) the position of the medial phalanx is usually aligned with the long axis of the proximal phalanx (e.g., Coombs 1978, 1979; Coombs and Rothschild 1999; Roussiakis and Theodorou 2001). In contrast, in Hö 142 the medial phalanx is positioned obliquely towards the ventral side (Fig. 1F), which seems to be similar to the anatomical position in chalicotheriines as illustrated by Zapfe (1979, fig. 147E). Lastly, the μCT scan of Hö 142 revealed that there is a prominent protrusion in the distal articulation between the bilateral keels of the proximal phalanx (Fig. 2A). Such a feature is not present in any schizotheriine (i.e., Coombs 1978, 1979; Kampouridis et al. 2023). However, Zapfe (1989) specifically discusses the presence of this feature in the phalanges of *Anisodon grande* from Devínska Nová Ves and it is also present in the proximal phalanx of *Chalicotherium goldfussi* (HLMD-DIN-3170) from its type locality Eppelsheim. Therefore, an attribution of Hö 142 to a chalicotheriine and not a schizotheriine is well supported.

## Interpretation of the ankylosis in Hö 142

The fusion of the first two phalanges, as seen in Hö 142, is not observed in any other chalicotheriine specimen, despite the large collections of phalanges belonging to this group from localities like the Middle Miocene Sansan in France and Devínska Nová Ves in Slovakia (e.g., Zapfe 1979; Guérin 2012). This is in stark contrast to the commonly observed non-pathological fusion of phalanges in some schizotheriines (Coombs and Rothschild 1999). Coombs and Rothschild (1999) conducted the only detailed study of the phalangeal fusion seen in the schizotheriines *Moropus*, *Tylocephalonyx*, *Phyllotillon*, *Metaschizotherium*, and *Ancylotherium* (e.g., Holland and Peterson 1914; Butler 1965; Coombs 1979, 2009; Coombs and Rothschild 1999; Roussiakis and Theodorou 2001; Fahlke and Coombs 2009; Heissig and Fejfar 2013). They acquired radiographs and sagittal sections of several duplexes of the North American representatives *Moropus* and *Tylocephalonyx*, allowing them to study the internal anatomy of the bones (Coombs and Rothschild 1999). They observed the resorption of cortical bone and the intertwining of the trabeculae between the fused phalanges (Coombs and Rothschild 1999). While the cortical resorption and the trabecular bridging were present in all specimens, their degree varied significantly depending on the specimen, the region within the bone (i.e., dorsal vs. ventral), and even the exact position of the section (Coombs and Rothschild 1999).

In the duplexes of the schizotheriines *Metaschizotherium fraasi* (SMNS-P-5815) and *Ancylotherium pentelicum* (NHMW-GEO-2019/0098/0007) we also observed the resorption of cortical bone in the area of the fusion, with only little cortical bone being left on the ventral side of the proximal phalanx (cb in Fig. 2B–C), whereas in the dorsal and central part of the fused area, the trabecular bone seems completely intertwined (tb in Fig. 2B–C), similar to what was observed in *Moropus* and *Tylocephalonyx* (Coombs and Rothschild 1999). In Hö 142, however, we do not see any resorption of the cortical bone (Fig. 2G). Instead of the cortical resorption, typical for physiological schizotheriine duplexes, the fusion zone in Hö 142 shows patches of endosteal new bone deposition (bn in Fig. 2F, H), consistent with a reactive peri-lesional response rather than normal remodeling. Trabecular voids adjacent to the fusion exhibit partial obliteration by woven bone neoformation (bn in Fig. 2H–I), indicative of localized inflammatory or mechanical insult rather than the uninterrupted trabecular interdigitation seen in physiological fusions. Bone neoformation is not observed in the non-pathologically fused duplexes in schizotheriines (Fig. 2B-C; Coombs and Rothschild 1999). A discrete dorsal exostotic outgrowth is present at the phalangeal junction in Hö 142 (Fig. 1B,K, 2A,F), which, per Rothschild et al. (2023, p. 49), most likely represents a reactive periosteal response rather than a normal morphological variant. On the ventral side of Hö 142, a larger area is covered by exostoses, which aligns with a severity of rank 3, based on the osteopathology scale developed for fossil and extant rhinos proposed by Stilson et al. (“clear protrusion” in 2016, fig 2.) and tentatively applied here on a chalicothere. Because physiological schizotheriine duplexes lack both extensive woven-bone infilling and prominent exostoses (Coombs and Rothschild 1999; Rothschild et al. 2023), the suite of reactive features in Hö 142 supports a pathological ankylosis rather than an evolutionary synostosis. Therefore, the fusion seen in Hö 142 is considered to be pathological and not physiological.

## Discussion

The study of paleopathologies can provide valuable information about the ecology of extinct organisms, including their lifestyle and interaction with their environment (e.g., Iliopoulos 2003; Böhmer and Rössner 2018; Gutherz et al. 2020; Cruzado-Caballero et al. 2021). The herein reported pathological fusion of proximal and medial phalanges in a chalicotheriine and the associated immobilization of the digit mirrors the physiological condition observed in schizotheriines (Coombs and Rothschild 1999). This peculiar mimicry provides a unique window into the development and functionality of the parallel evolution of restricting the digit mobility, through interphalangeal ankylosis in Schizotheriinae and reactive notched-joint formation in Chalicotheriinae. Most Schizotheriinae restricted the movement of the interphalangeal joint between the proximal and the medial phalanx (e.g., Gaudry 1862; Holland and Peterson 1914; Koenigswald (1932); Schaub 1943; Coombs 1978, 1979, 2009); Coombs and Rothschild 1999; Roussiakis and Theodorou 2001; Fahlke and Coombs 2009) by reducing the curvature of the bilateral keels of the proximal phalanx. The distal articulation of the proximal phalanx became flatter and the proximal articulation of the medial phalanx less concave, thereby reducing the possible angle at which the phalanges can move. In digit II of the manus, and sometimes also in the pes, the two phalanges actually completely co-ossify, forming the duplex bone, which completely immobilizes the joint. Chalicotheriinae, on the other hand, restricted the movement of the interphalangeal joint by creating a specialized notched joint between the phalanges. This feature consists of a protrusion in the distal articulation of the proximal phalanx, positioned between the bilateral keels and a corresponding notch on the proximal articulation of the medial phalanx, located along the central keel (Fig. 2A). Together, these structures greatly restrict movement at the joint, rendering it nearly immobile. Therefore, members of both subfamilies achieved a similar functional result – i.e., the restriction of movement or even immobilization of the interphalangeal joint – by the acquisition of different traits.

The mechanisms of how these modifications were acquired may be related to increased stress levels on the ligament capsule in the joint area (Coombs and Rothschild 1999, p. 689), possibly associated with the usage of the large claws. This stress could also be the cause of the fused duplex in schizotheriines and explain the fracture seen in specimen UCMP 14377 of *Moropus elatus* (Coombs and Rothschild 1999, fig. 5). The same joint stress could also be related to how the notched joint was acquired in chalicotheriines, as the restriction of anteromedial movement that is related to the notched joint may stabilize the digit and overall dampen the forces that are applied to it. Increased stress levels in this joint area – although we do not exclude some kind of disease or inflammation that could have affected the bone – may also have been the cause for the ankylosis in Hö 142. The comparison of the function of the interphalangeal joints in both schizotheriines and chalicotheriines (including the osteopathology in Hö 142) provides evidence for a curious case of parallel evolution, where both subfamilies achieved the restriction of the movement or even immobilization of the interphalangeal joint by the acquisition of different traits. In schizotheriines we see the fusion of the proximal and medial phalanx, whereas in chalicotheriines a notched joint is formed that greatly limits the mobility of the digit.

## Data Availability

The CT-scans of Hö 142 and SMNS-P-5815 are available on Morphosource (https://www.morphosource.org/) under the Project ID 000769060. The CT-scan of NHMW-GEO-2019/0098/0007 is available on the NHMW Data Repository: 10.57756/b1su1x.
